# Erythema multiforme induced by clindamycin diagnosed by patch test

**DOI:** 10.1186/1939-4551-8-S1-A169

**Published:** 2015-04-08

**Authors:** Bruna Gama Saliba, Nathalia Pessoa Simis, Marisa Rosimeire Ribeiro, Laila Sabino Garro, Nathália Coelho Portilho, Jorge Kalil, Pedro Giavina-Bianchi, Antonio Abílio Motta, Marcelo Vivolo Aun, Violeta Regnier Galvão

**Affiliations:** 1 University of São Paulo, Brazil; 2 Brigham and Women's Hospital, Harvard Medical School, USA

## Background

Erythema multiforme (EM) is a skin disorder most commonly caused by herpes virus infection, but drugs can also be involved. We report a patient who had developed a EM due to clindamycin and the diagnosis was confirmed with a skin patch test.

## Methods

Literature review and case report.

## Results

A 17 years of age male was admitted in a University Hospital In São Paulo, Brazil, because he had been a victim of a car accident in May 2012. He suffered a tibia open fracture and was submitted to a surgical treatment. Three days after the procedure he developed face rash, cutaneous itching, target lesions in oropharynx and lower limbs peeling. He was being treated with Clindamycin, Ciprofloxacin, Dipyrone, Ketoprofen and Tramadol. The patient evolved with fever and leucocytosis, without eosinophilia. This reaction was diagnosed as EM major by Dermatology Unit and he was successfully treated with antihistamines and corticosteroids, besides suspected drugs substitution. After been discharged the patient was referred to the Allergy Unit to perform a drug hypersensitivity investigation. He was submitted to patch test with all the suspected drugs diluted in petrolatum 10%. Only the clindamycin patch test was positive, which was confirmed with a second patch test. The patient also presented reactivation of previous lesions.

## Conclusions

As far as we know, this is the first patient who had developed erythema multiforme due to clindamycin. The patch test was essential to confirm the diagnosis and the use of all other drugs which were present at the time of the reaction could be released.

## Consent

Written informed consent was obtained from the patient for publication of this abstract and any accompanying images. A copy of the written consent is available for review by the Editor of this journal.

